# Patient and general practitioner experiences of implementing a medication review intervention in older people with multimorbidity: Process evaluation of the SPPiRE trial

**DOI:** 10.1111/hex.13630

**Published:** 2022-10-17

**Authors:** Caroline McCarthy, Ivana Pericin, Susan M. Smith, Bridget Kiely, Frank Moriarty, Emma Wallace, Barbara Clyne

**Affiliations:** ^1^ Department of General Practice, HRB Centre for Primary Care Research RCSI University of Medicine and Health Sciences Dublin Ireland; ^2^ School of Social Work and Social Policy Trinity College Dublin Dublin Ireland; ^3^ Department of Public Health and Primary Care Trinity College Dublin Dublin Ireland; ^4^ School of Pharmacy and Biomolecular Sciences, Royal College of Surgeons in Ireland University of Medicine and Health Sciences Dublin Ireland

**Keywords:** deprescribing, multimorbidity, polypharmacy, potentially inappropriate prescribing, process evaluation

## Abstract

**Introduction:**

The SPPiRE cluster randomized controlled trial found that a general practitioner (GP)‐delivered medication review that incorporated screening for potentially inappropriate prescriptions (PIP), a brown bag review and a patient priority assessment, resulted in a significant but small reduction in the number of medicines and no significant reduction in PIP. This process evaluation aims to explore the experiences of GPs and patients and the potential for system‐wide implementation.

**Methods:**

The trial included 51 general practices and 404 participants with multimorbidity aged ≥65 years, prescribed ≥15 medicines. The process evaluation used mixed methods and ran parallel to the trial. Quantitative data was collected from the SPPiRE intervention website and analysed descriptively. Qualitative data on medication changes were collected from intervention GPs (18/26) and a purposive sample of intervention patients (27/208) via semi‐structured telephone interviews. All interviews were transcribed verbatim and analysed using a thematic analysis. Qualitative and quantitative data were integrated using a triangulation protocol.

**Results:**

The analysis generated two themes, intervention implementation and mechanisms of action, and both were underpinned by the theme of context. Intervention delivery varied among practices and 45 patients (28%) had no review, primarily due to insufficient GP time. 80% of reviewed patients had ≥1 PIP identified, 59% had ≥1 problem identified during the brown bag review and 79% had ≥1 priority recorded. The brown bag review resulted in the most deprescription of medications. GPs and patients responded positively to the intervention but most GPs did not engage with the patient priority‐setting process. GPs identified a lack of integration into practice software and resources as barriers to future implementation.

**Conclusion:**

The SPPiRE intervention had a small effect in reducing the number of medicines and this was primarily mediated through the brown bag review. The context of resource shortages and deep‐seated views around medical decision‐making influenced intervention implementation.

**Patient or Public Contribution:**

Qualitative data on the implementation of the medication review and their wider views on their medicines was collected from older people with multimorbidity through semi‐structured telephone interviews.

**Clinical Trial Registration:**

The SPPiRE trial was registered prospectively on the ISRCTN registry (ISRCTN12752680).

## 1 INTRODUCTION

There is a growing population of older people living with multiple chronic conditions or multimorbidity.^1^ Prescribing for patients with multimorbidity, especially those with more significant polypharmacy can be risky due to potential drug‐drug and drug‐disease interactions.^2^ The application of multiple single disease guidelines to an individual person with multimorbidity is often not feasible or advisable and can lead to an unacceptable treatment burden.^3^ Multimorbidity and polypharmacy guidelines advise identifying patients at risk of medication‐related harm, screening for potentially inappropriate prescriptions (PIP) and tailoring care to individual patient priorities.^4^, ^5^, ^6^ However a 2019 overview of multimorbidity and polypharmacy guidelines recognized that despite these guiding principles of tailoring care, specific recommendations are often missing.^7^

The Supporting Prescribing in Older Patients with Multimorbidity in Primary Care (SPPiRE) cluster randomized controlled trial (RCT) demonstrated that the SPPiRE intervention was effective in reducing the number of medicines but did not demonstrate an effect on PIP, in patients aged ≥65 years and prescribed ≥15 repeat medicines in Irish primary care.^8^ The development of the SPPiRE intervention is described in detail elsewhere.^9^ The SPPiRE intervention comprised professional training in the form of online training videos and a web‐guided medication review, where general practitioners (GPs) were supported in identifying PIP and guided to perform a brown bag medication review (where the patient brings in all their medicines and each is reviewed in turn), and assess patient treatment priorities. Although commonplace in other jurisdictions,^10^ nonmedical prescribing is currently not in place in Irish primary care and so the SPPiRE intervention was targeted at GPs. The SPPiRE intervention was a complex pragmatic intervention as it had a number of interacting components and a degree of flexibility in how it was delivered.^11^ As recommended by the Medical Research Council, as part of their framework on complex interventions, a process evaluation was performed alongside the effectiveness evaluation of the SPPiRE intervention, to assess how the intervention was implemented and resulted in change and how participants (both GPs and patients) responded to it.^12^

The overall aim of the SPPiRE process evaluation was to assess the potential for system‐wide implementation. Objectives included exploration of intervention implementation and the mechanism of action of the intervention. Specifically; what were the effective and ineffective components of the intervention, how did GPs and patients respond to it, and how did the overall context affect intervention implementation?

## 2 METHODS

The methods for this mixed methods parallel process evaluation have been described in the published protocol,^13^ and were developed based on a framework that was designed to guide the conduct of process evaluations for cluster RCTs.^14^ Quantitative and qualitative data collection techniques were employed and data was analysed in isolation and integrated using triangulation to gain a comprehensive understanding of intervention implementation and mechanism of action. Ethical approval was granted by the Irish College of General Practitioners Research and Ethics Committee and all recruited GPs and patients gave fully informed written consent. A convergent parallel mixed methods design was adopted.

### 2.1 Intervention description

The SPPiRE intervention comprised professional training in the form of online videos and a web‐guided medication review. Intervention GPs were asked to book a double appointment and ask each patient to bring all their medicines in with them for their SPPiRE review. The medication review process was guided by the SPPiRE website where GPs were:

1. supported in identifying PIP (from a predefined list of 34 indicators based predominantly on the STOPP/START version 2 criteria,^15^ see Supporting Information: Appendix 1),
2. provided with suggested treatment alternatives for identified PIP,
3. prompted to perform a brown bag medication review and record any problems identified and,
4. prompted to assess and record patients’ priorities for treatment.

**Figure 1:**
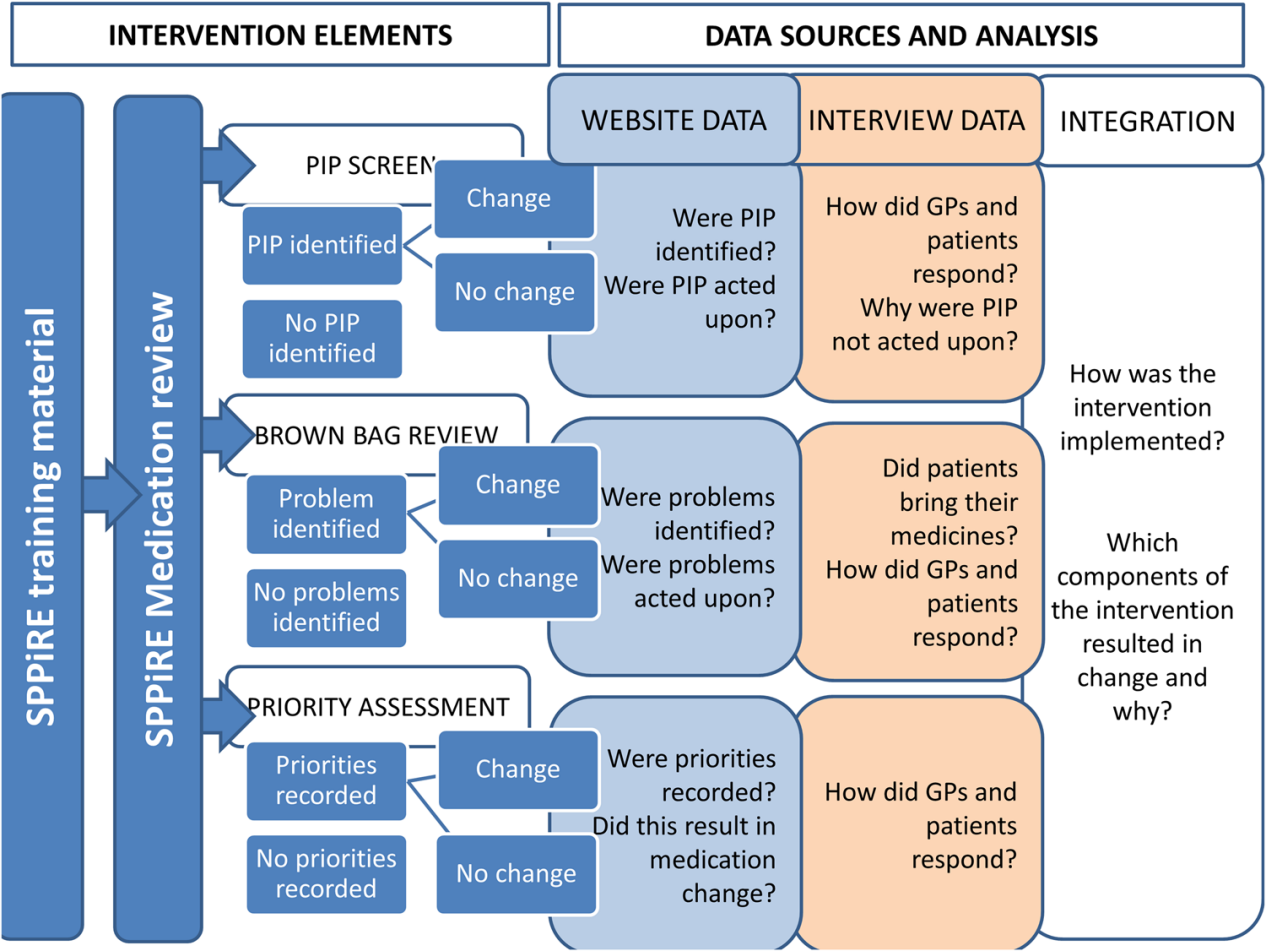
Hypothesized mechanism of action for SPPIRE intervention

Figure 1 illustrates the hypothesized mechanism of action of the intervention and how this was tested.

### 2.2 Study population

The SPPiRE study was conducted in 51 general practices throughout the Republic of Ireland, which identified and recruited 404 patients aged ≥65 years and prescribed ≥15 repeat medicines, see Supporting Information: 1 for an illustration of the methods and results of the cluster RCT. Twenty‐six practices (208 patients) were allocated to the intervention group and 25 practices (196 patients) to control. Given that the primary aim of the process evaluation was to assess intervention implementation, our target population for this process evaluation was intervention arm participants only. All intervention GPs were invited to take part in semi‐structured telephone interviews and 18 of 26 consented. Intervention patients were purposively sampled to include a mix of male and female patients and those who had some and no medication changes. Forty‐six patients were telephoned and invited to take part in semistructured telephone interviews, 11 declined citing fatigue or hearing difficulties and 8 did not reply. Twenty‐seven patients consented to participate in an interview (58% of those invited) and were interviewed.

### 2.3 Data collection

To address the objectives, quantitative and qualitative data sources were used.

Quantitative data was collected from the SPPiRE website for all intervention patients who had a SPPiRE medication review. GPs inputted PIP data, and medication‐related concerns during the brown bag review and recorded patient treatment priorities. For each PIP, concern or priority identified the GP was prompted to discuss and record the outcome (which included no action). GPs also recorded an immediate pre‐ and postintervention medication count. Quantitative data was also collected from a general practice profile questionnaire which was submitted by all recruited practices and included details on practice demographics and repeat prescribing systems. Quantitative data on patient demographics were collected from a self‐administered postal questionnaire. Quantitative data from the main trial outcomes was used to compare PIP prevalence.

Qualitative data were collected using semi‐structured telephone interviews and study manager logs of patient and practice contact during recruitment, intervention delivery and follow‐up. The GP interview topic guide focused on pre‐intervention prescribing practices, intervention implementation and overall views on participation (see Supporting Information: Appendix 2). The patient interview topic guide focused on attitudes towards medicines and experience of the SPPiRE medication review (see Supporting Information: Appendix 3). Interviews were conducted by CMC and BK, who a female and GPs by professional background, and were trained by BC, a female senior researcher with experience in qualitative methods.

### 2.4 Data analysis

Quantitative data were analysed using descriptive statistics in Stata version 17 by CMC. Outcomes of interest were the number of medicines pre‐ and postreview, the number of PIP identified and outcome of identification, and the number of concerns and priorities identified and the outcome of identification. This data was collected from the SPPiRE intervention website and expressed as counts, frequencies and proportions, and mean reduction in the number of medicines per patient post review by practice.

Telephone interviews were all audiotaped, transcribed verbatim and uploaded into NVIVO 12 to perform a thematic analysis.^16^ Interview audio files and transcripts were listened to and read repeatedly by two researchers (IP and CMC) to ensure familiarization with the data. Codes were generated both inductively from recurring themes in the data and deductively using the four Normalization Process Theory (NPT) constructs to describe implementation.^17^ NPT is a contemporary social theory that has been used to understand the factors in intervention implementation and has four constructs; coherence, cognitive participation, collective action and reflexive monitoring. The initial codes were developed by a researcher who was independent from the main trial (IP) and by another researcher, who was also the study manager (CMC). This combination of researchers captured both emic (insider) and etic (outsider) perspectives and provided for a rich analysis of the data.^18^ Codes were then labelled and organized into major subcategories and categories, which were then organized into the main themes. The final categories and themes were reviewed, discussed and agreed upon by all members of the process evaluation study team. Participating practices were coded by number for example GP1, and their participating patients were coded using the practice code with a patient code, for example, GP1P1.

### 2.5 Integration

Qualitative and quantitative data were integrated using a triangulation protocol.^19^ Data were initially analysed in isolation and during the integration process identified themes from qualitative data and quantitative results were further explored from the alternative data sources and the relationship between the two data sources was coded as either being in agreement, partial agreement, silent or dissonant.

## 3 RESULTS

The analysis generated two themes, intervention implementation and mechanisms of action, both of which were underpinned by the third theme of context.

### 3.1 Characteristics of the sample

Practices self‐classified as either urban, rural or mixed and were categorized into three groups based on the size of the practice. There were 14 urban (54%), 4 rural (15%) and 8 mixed practices (31%). Of the 18 intervention GPs interviewed, 11 were male, 10 were from urban, five were from mixed and three were from rural practices (see Supporting Information: Appendix 4). In Ireland, GPs are independent contractors but sometimes employ other GPs as assistants. Of the interviewed GPs, two were assistants and the remaining 16 were either the sole principal GP or in partnership with other GPs in the practice. GPs who declined or did not respond to telephone interview invitations had similar characteristics to those that participated in an interview but were over‐represented by practices that did not perform the medication reviews (see Supporting Information: Appendix 5). Of the 27 intervention patients interviewed 12 were male, the mean age was 73.7 years (SD, 5.4) and this was slightly younger than the mean age for all intervention patients at baseline (76.7 years) (see Supporting Information: Appendix 4). Patient interviews lasted an average of 11.0 minutes (min 4.0–max 25.9), while GP interviews lasted an average of 19.5 minutes (min 11.3–max 32.1). Looking at the immediate post‐intervention medication count recorded by GPs, interviewed patients had on average 1.59 medicines stopped (SD, 2.50) compared with 1.71 medicines in all reviewed patients (SD, 2.31).

### 3.2 Intervention implementation

#### 3.2.1 Response of practices: How the intervention was adopted

Between 2 January 2018 and 11 May 2020, 163 of 208 (78%) intervention patients had a SPPiRE medication review. Intervention practices were given 6 months from the date of allocation to complete all medication reviews. Eleven practices completed the medication review within this time frame (see Supporting Information: Appendix 5) and the remainder were on average 7 weeks late in completing the intervention (range of 20 weeks early and 47 weeks late). The most commonly reported reasons for these delays were capacity related, that is, practice time constraints and staffing issues. Overall, a higher proportion of participants from smaller and rural practices had their SPPiRE review within the designated time frame (see Supporting Information: Appendix 5), however, there was significant variation across practices and a small number of practices in the rural category.

#### 3.2.2 Delivery to individuals (patients)

**Figure 2:**
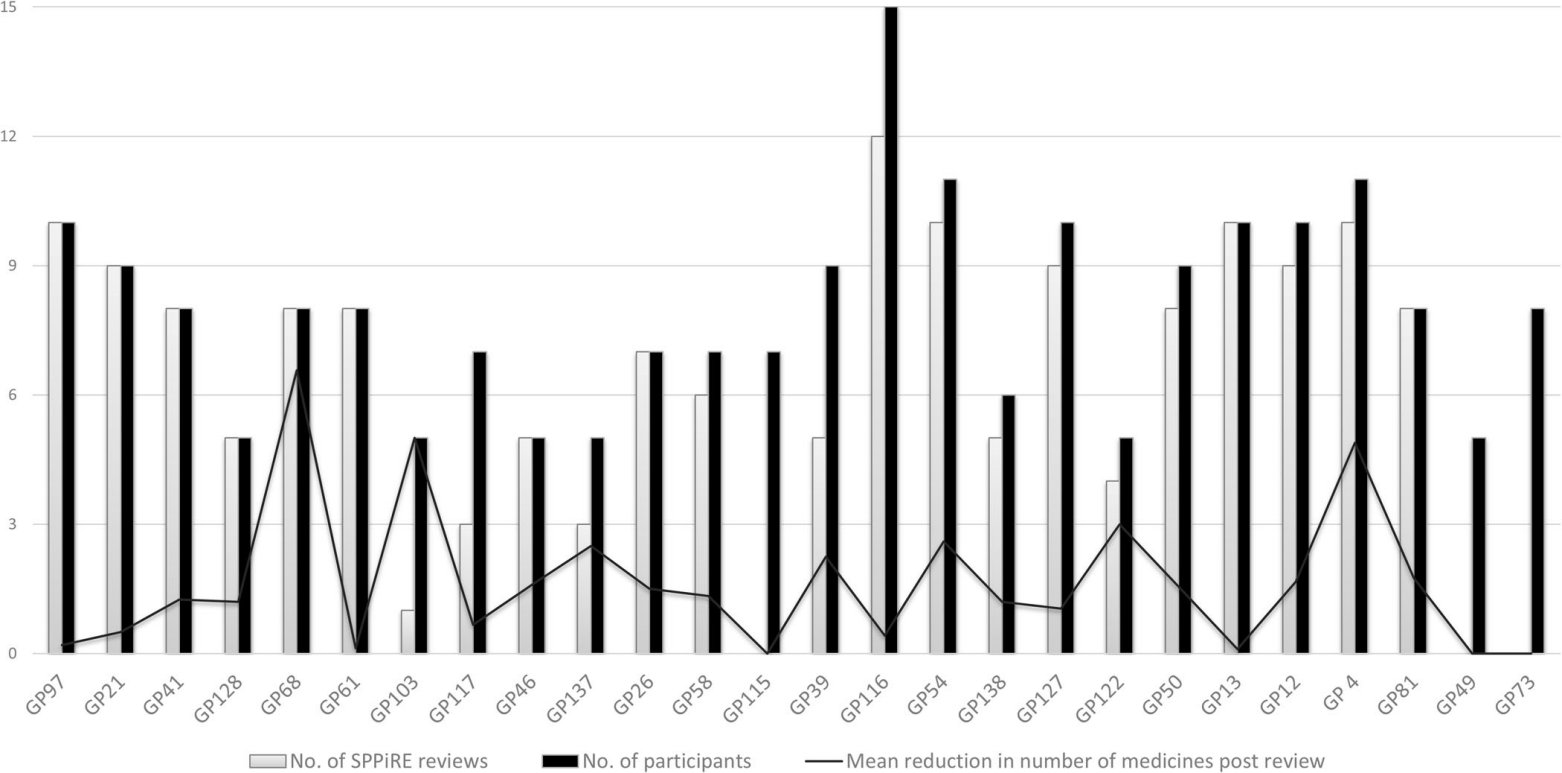
Mean reduction in number of medicines per practice postintervention

Data from the study research logs highlighted that of the 45 patients who did not have a review, 38 were because the GP reported they did not have enough time to complete the review(s). Three patients did not have a review because they had died and four were either too unwell or in hospital and thus unable to attend for a review. Figure 2 illustrates the number of participants per practice who had a SPPiRE medication review and the immediate mean reduction in the number of medicines per practice, illustrating the intra‐practice variation in deprescribing. Included in the 45 patients who did not have a review, were 20 patients from three intervention practices who did not perform any reviews within the specified time, the GPs again cited time constraints and staffing issues as the reasons.

#### 3.2.3 Response of participants to the intervention

In terms of the NPT construct coherence (sense‐making of the intervention), 7 of the 27 interviewed patients had difficulty remembering their review as separate or different to routine care. Patients described significant treatment burden and recounted instances of medicines being stopped in hospital clinics or during admissions, and so might have had difficulty differentiating SPPiRE from other care. On the other hand, GPs generally saw the SPPiRE review process as separate from routine care, and this was viewed positively by some ‘it felt like a luxury that you could sit down in practice and take the time to go through it’ (GP61, female, GP assistant). Despite viewing SPPiRE as separate from routine care most GPs made few changes or adaptations to the rest of their practice to incorporate reviews and frequently participated in SPPiRE in isolation from the rest of their practice activity, see Table 1 for illustrative quotations. For example, two GPs described coming in on days off or after hours to complete the reviews as they felt this was the only way they could get them done. In terms of collective action (practical work of doing the intervention), all GPs identified PIP and most reviewed the patients’ notes before the face‐to‐face patient consultation, ‘just to familiarize ourselves again with all the past medical history and try and see what the indication was for each of the medications’ (GP26, female, GP partner). Adherence to the brown bag review and assessment of patient priorities processes varied. Although GPs reported finding the brown bag review process useful, the majority of interviewed patients did not bring all their medicines in with them ‘I thought if it was on the list, there wasn't much point in carrying it in…because everything is on the computer’ (GP39P41, female, 74 years). Of the 23 intervention practices that implemented either some or all of the reviews, 11 recorded at least one treatment priority for all patients, in the remaining 12 practices the proportion of patients with at least one recorded priority varied from 30% to 88%. Overall patients and GPs were positive in their appraisal and evaluation of the intervention (reflexive monitoring), ‘There was nothing I didn't like about it, I felt it was useful…it was generally useful and informative’ (GP26P6, male, 75 years), but GPs felt adequate resourcing and integration with practice electronic health record systems would be vital for system‐wide implementation.

**Table 1:** Thematic analysis of SPPiRE medication review implementation using NPT

NPT Core Construct	Construct components	Example quotes
*Coherence* Making sense of the intervention	GPs saw the SPPiRE review as a *separate process to routine care* that would be beneficial	I suppose these are patients we were seeing them anyway, so it wasn't as if we were creating extra appointments trying to get them back in again…. But we're seeing them when we were forearmed, I guess forewarned with other information, with stuff that we could be doing better or mistakes that were being made. (GP21, male, GP partner) It was really good and again, it felt like a luxury that you could sit down in practice and take the time to go through it. (GP61, female, GP assistant)
	GPs often *took on the process alone*; only 6 interviewed GPs said that other GPs from the practice were involved	…it wasn't that they were antagonistic. When the proposal came in and we brought it up at the practice (meeting) and said…we'd all like to do that. But like with the best will in the world it just didn't get communicated to everybody. (GP39, male, GP partner)
	GPs felt their *role was central* to ensuring safe prescribing for these patients	I suppose maybe we're the only central person to their prescriptions. (GP12, female, GP partner) I mean it's one of the most important, one of the most important aspects of our role, and the most medico‐legal aspect of our role (GP46, male GP partner)
*Cognitive Participation* Involvement with the intervention	*Adaptation of the practice* varied. Practices were advised to book double time slots for SPPiRE reviews.	I actually did it outside of hours…so it didn't really impact and probably is a bit artificial but they didn't really impact on you know, day‐to‐day working because I wanted to give it enough time. (GP4, male, GP partner) So I ended up just going in on time off and doing it and then you can just kind of dedicate a couple of sessions to doing it and getting it done properly that way (GP61, female, GP assistant) …we had to make sure that when they made their appointment that the receptionist knew that it was actually for the study and that they gave a half an hour doctor appointment so that we had enough time. (GP26, female, GP partner) The practice manager giving me protected time to complete it. (GP54, male, GP partner)
	Positive experience with the *training videos*, particularly that they were short and easy to access	…the training materials were very good…having everything in one place and links to points on the online reviews was just invaluable. You know you could find the stuff you needed to make a decision quickly. (GP128, female, GP partner) They were actually very good. And so would take from this is that I would probably run them now as a teaching session in the practice…and suggest that this is what we do and this is how we do a medication review and that we plan to do it in a more structured way (GP39, male, GP partner) I thought they were really good. They weren't too long which was great, so it was easy to get through them quite quickly and they were to the point. (GP26, female, GP partner)
*Collective action* How practical work of doing the intervention is carried out	*Familiarization with the notes* and medicines before the review	I ran the SPPiRE thing first before they come in, so that is at least flagged up what issues there might be with their prescription. (GP39, male, GP partner) Obviously I did a review of their medications first; checking for interactions, checking for were they on the appropriate dose, checking that they were being monitored correctly, and that you know things were co prescribed appropriately. (GP12, female, GP partner) Before we called the patient in we would have had a good look through the file just to familiarize ourselves again with all the past medical history and try and see what the indication was for each of the medications. (GP26, female, GP partner)
	*Adherence to the SPPiRE review process*: most patients did not bring their medicines with them, half of GPs felt including patient priorities was either challenging or not helpful	Not the actual medicines, no. I would have had a list. Well, the doctor had a list of them anyhow. (GP54P28, male, 70 years) Yeah, I sort of got the feeling they didn't really know what I meant or and maybe I didn't word it very well. (GP128, female, GP partner)
*Reflexive Monitoring* Evaluation and appraisal of the intervention	GPs felt they had a *better awareness of PIP*, were more *confident stopping medicines* but had not anticipated the *amount of time* involved	I found it…educational because a lot of stuff they had mentioned in terms of drugs to look out for maybe I wasn't familiar with or wasn't doing it on a day to day basis, that then prompted how I would prescribe in the future if that makes sense. (GP4, male, GP partner) I suppose I think since I did the SPPiRE study I have probably become a lot more aware of trying to stop things in elderly particularly. (GP26, female, GP partner) It does take up more sort of energy and time then we may have anticipated. (GP54, male, GP partner)
	Patient's generally felt *reassured* and *better informed* about their medicines	There was nothing I didn't like about it, I felt it was useful…it was generally useful and informative. (GP26P6, male, 75 years)) I suppose just to be able to chat to him about my concerns…it made me feel more comfortable taking them because I knew exactly, what was what. (GP41P8, female, 68 years)
	Suggested solutions and improvements	…perhaps asking them what your priorities are earlier before they even came into you, instead of just springing it on them in the consultation would have proven more useful. (GP4, male, GP partner) I suppose if people got an extra fee for it that would always be an incentive you know yourself. (GP137, female, GP partner) I think it would have to be something that's integrated into our software system that we work from. (GP12, female, GP partner) I sometimes find the patients are more open with the practice nurse. The doctors, the prescribers are the experts. (GP137, female, GP partner)

Abbreviation: NPT, Normalization Process Theory.

### 3.3 Mechanism of action of the intervention

The SPPiRE intervention was effective in reducing the number of medicines but had no effect on PIP outcomes.^8^ Practice characteristics that might influence the mechanism of action such as size and location were included as covariates in the trial analyses and did not significantly influence the results.^8^ In terms of the effect of practice organization, a sensitivity analysis indicated that there was no difference in the results when ‘presence of a written practice repeat prescribing policy’ was included as a covariate.^8^ Including patient factors such as patients’ attitudes to deprescribing^20^ also had no significant effect on the results.

#### 3.3.1 Attitudes towards medicines and deprescribing

Whether a medicine was stopped or changed depended on multiple factors including the patients’ attitude, the medicine and the doctor‐patient relationship. Most patient interviewees expressed they were open to stopping medicines but there was reluctance or fear in stopping some medicines, particularly those that had been prescribed for a long time, ‘It's funny when you're taking tablets and if it's working for you. It's like everything else, if it works don't break it. You know what I mean, you're afraid’ (GP50P1, female, 68 years). In particular, there was a reluctance to discontinue benzodiazepines, ‘The only category that they're not always happy with is the benzos because they're so …used to those benzos and sleeping tablets’ (GP97, male, GP partner). With respect to their SPPiRE review most patients who described a change during their review viewed this positively, even in an instance where the change did not work out and a medicine that had been stopped had to be restarted; ‘…I feel fine about it. They did their best to try to reduce my medication, which is a good thing. The less you take, the less side effects you're going to have. So I was all for it’ (GP4P13, female, 73 years). Most patients were well‐known to the GP however, five GPs described doing reviews with patients not well‐known to them and most felt this made the process more complicated. Another factor that influenced the decision to stop medicines was the complexity of the patient; ‘She's a complex patient who you tweak rather than make any real, you tweak to keep her propped up really’ (GP137, female, GP partner). Patients’ background views on their medicines varied with four patients being wary of too many medicines and others who were hoping for a ‘miracle medicine’ (GP122P11, female, 76 years). Patients’ views also varied in how involved they liked to be in making decisions about their medicines. Some patients described being wary of ‘pill popper’ (GP50P1, female, 68 years) or doctors ‘adding on something all the time’ (GP39P41, female 74 years). But many described complete and unquestioning trust in their doctor, ‘Whatever I'm ordered to take, I take and that's it’ (GP54P25, female, 84 years). Some GPs described how this trust negatively impacted shared decision making, ‘…. they tend to be more respectful…respect in a negative way if you know what I mean, they tend not to be questioning. Which is not what you want’ (GP137, female, GP partner). The SPPiRE intervention facilitated a discussion and change in these instances, to ‘intervene and be proactive about it’ (GP21, male, GP partner).

#### 3.3.2 Recorded medication changes

With respect to changes in prescribing activity recorded on the SPPiRE website, GPs identified 291 PIP in the 163 patients who had a review (1.79 PIP per person), resulting in 145 different medication changes and 11 referrals for blood monitoring. The same PIP criteria were used in the intervention and for trial outcomes and GPs identified less PIP compared with the baseline assessment of intervention prescriptions (*n* = 208) by the trial's blinded pharmacist, who identified 517 PIP (2.49 PIP per person). When compared with the baseline numbers in the 163 participants who had a review there was a total of 410 PIP (2.52 per person). There were also differences in the proportions of PIP identified, with GPs less commonly identifying proton pump inhibitor (PPI) and anticholinergic‐related PIP, see Supporting Information: Appendix 6 for the numbers and proportions of the top 10 identified PIP. The website also captured data on the outcome of identified PIP. Overall the identification of a PIP resulted in either no change (31%) or stopping a medication (21%) the majority of times (see Supporting Information: Appendix 7). For PPIs, the most common outcome was a dose change (45%) and for benzodiazepines was no change (53%), consistent with the qualitative findings. The prescription of an anticholinergic medicine with a specific comorbidity resulted in no change in 50% of identified cases. On further review of website data, the PIP was frequently an antimuscarinic inhaler which GPs may have judged to be less likely to cause systemic side effects. In at least one other case the PIP was a urinary anti‐spasmodic, initiated in secondary care for urgency symptoms secondary to benign prostatic hypertrophy. The qualitative findings indicated that patient preferences, the complexity of the patient and how familiar the GP was with the patient's history all influenced the GPs’ decisions on whether to make changes.

With respect to the brown bag review, a total of 237 different medication concerns in 95 patients (58%) were recorded. Examples of concerns included non‐adherence to a medicine, side effects and monitoring issues and these were identified by either the patient or GP. The most common outcome for identified concerns was stopping a medicine (41%), (see Supporting Information: Appendix 8 for outcomes of identified medication‐related concerns). As previously described, the majority of interviewed patients reported not bringing their medicines to the review. However, most GPs found this process useful and saw it as an opportunity to reflect on treating these complex patients in a different way, ‘I think the learning for me is that you know people get stuck on, get put on tablets and sometimes haven't the vaguest of ideas of what they're taking them for, you know?’ (GP58, male, GP partner). In instances where the patient did not bring their medicines, interviewed GPs described working off a list instead but the interview data indicated that this process was more effective when the patient brought their medicines with them; ‘I found one or two that were on a list, on a drug that they're getting a prescription for but they haven't taken it for months’ (GP39, male, GP partner). It was not possible to ascertain from the quantitative data if more changes were made when the patient had brought the medicines in with them.

**Figure 3:**
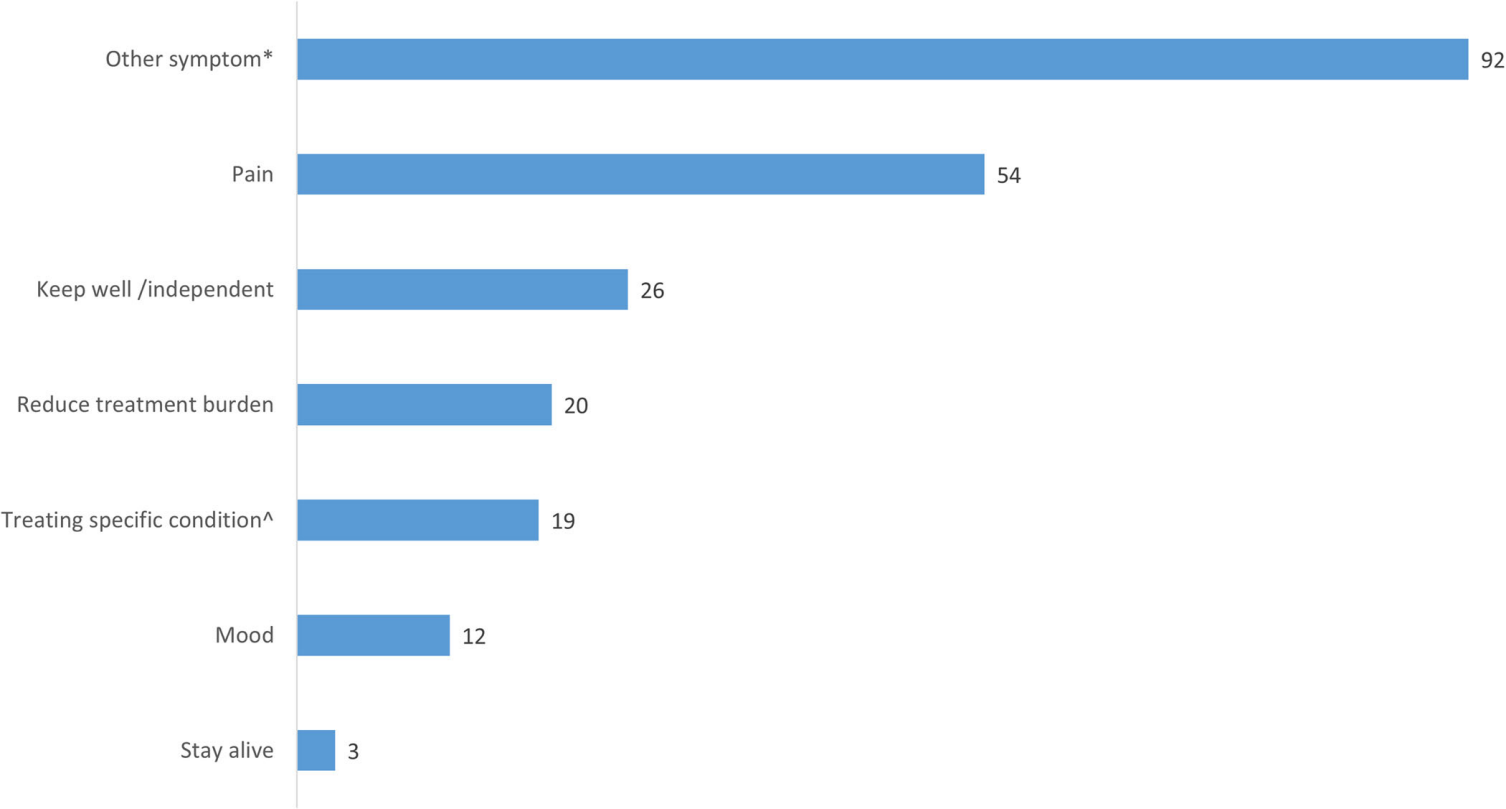
Patient treatment priorities recorded by GP. ^Examples included Type 2 Diabetes Mellitus targets, blood pressure targets and avoiding a specific outcome such as another heart attack or stroke. *Common examples included constipation, diarrhoea, urinary incontinence, fatigue, shortness of breath, insomnia and dizziness.

The final process prompted the GP to ask the patient about his or her priorities for treatment. GPs identified 223 different priorities in 128 patients (79%), most commonly treating pain and other symptoms, see Figure 2. Assessing treatment priorities resulted in a medication change for 51 of these patients, usually a dose change, see Figure 3.

#### 3.3.3 Attitudes to treatment priority assessment

GPs views on the elicitation of patient priorities varied considerably. Four of the 18 interviewed reported that they already knew their patients’ priorities and so they didn't need to ask, five found it challenging and said the patients struggled to understand the question or focused on multiple symptoms. Only three GPs reported finding this process useful, see Table 2. There is little data from patient interviews as many found it difficult to answer questions about their treatment priorities, ‘I don't understand what it's all about’ (GP4P13, female, 73 years). One patient, who previously worked as a healthcare professional spoke positively about the process, describing it as a way of ‘having an over look at the whole body, the whole beast’, whereas previously, ‘Nobody was looking at the big picture. I feel different people, different consultants prescribe different things’ (GP39P41, female, 74 years).

**Table 2:** GP responses to assessing patients’ treatment priorities

GP response	Example quotes
Unengaged (4)	I really didn't delve in too much for that. Some of them were probably incapable of understanding it or didn't want to understand it. (GP21, male, GP partner)
Useful (3)	All of these patients will be people we would in general tend to know very well. So we do make assumptions. So actually, getting them to verbalise what their priorities are is, I think, very useful. (GP122, male GP partner)
Not useful (4)	When you see a patient who's on a lot of medications, generally speaking that's because they've got quite a few medical problems and so therefore their priorities were sort of, you know, to have the medical problems addressed if possible. (GP58, male, GP partner)
Challenging (5)	Yeah, that was actually a bit tricky. I'm not sure, I didn't want to try and put words in their mouth. (GP128, female, GP partner)

#### 3.3.4 Integration of data

The overall context of resource shortage and GP and patient views on medical decision‐making affected how the intervention was implemented and exerted its effect. Table 3 integrates and summarizes the results of the qualitative and quantitative analyses with respect to the three identified themes.

**Table 3:** Integration of qualitative and quantitative analyses

Theme	Qualitative data	Quantitative data	Integration
Implementation	GPs saw SPPiRE as important and separate to routine care but there was little practice‐wide adaptation to fit medication reviews in	Nine of 26 intervention practices completed all reviews, and 11 completed their reviews within the allotted time frame. Over a fifth of participants had no medication review.	Agreement
Mechanism of action: PIP identification	GPs felt PIP identification was important GPs described feeling more confident in identifying PIP and stopping medicines	GPs identified less PIP than baseline pharmacist PIP identification resulted in change 55% of the time	Dissonance
Mechanism of action: Brown bag review	Not all patients brought their medicines in with them, although GPs thought this component was useful	Although concerns were only identified in less than 60% patients, this process resulted in the most deprescription of medicines	Partial agreement
Mechanism of action: Treatment priorities	A minority of GPs found this process helpful. There is little data from patient interviews as many found it difficult to answer questions about their treatment priorities.	Although almost 80% of participants had at least one priority recorded, this process rarely resulted in medication change	Agreement
Context	Staff and resource shortage affected implementation Deep seated views on medical decision making influenced engagement with components of the review	No contributing data	Silent

## 4 DISCUSSION

### 4.1 Summary of findings

This mixed‐methods process evaluation revealed that the SPPiRE intervention was implemented as intended in the majority of practices. A core component of the intervention involved GPs identifying PIP in patients. GPs, however, identified less PIP than anticipated by the study team and acted on them approximately half of the time, despite describing this process as important and valuable in interviews.

The brown bag review, where GPs reviewed each medicine with their patient and recorded any concerns identified either by the patient or GP, resulted in the most medication changes out of the various components of the review (146 medication changes related to 237 identified concerns). Most of the time this was stopping a medicine, suggesting that the intervention effect may have been mediated mostly through this process and is consistent with the overall trial results where there was evidence of an effect on the number of medicines, but not PIPs.^8^ Patient treatment priorities were mostly symptom‐based, particularly treating pain and this process led to fewer medication changes than PIP identification and the brown bag review. Overall GPs and patients did not engage with this process due to deep‐seated views on medical decision‐making. Although training material was viewed positively as informative and succinct, the purpose and process of assessing patient treatment priorities may not have been adequately covered.

The overall context of staff and resource shortage affected intervention implementation, and GPs identified adequate resourcing and integration with practice systems as prerequisites for system‐wide implementation. The SPPiRE intervention had a small effect in reducing the number of medicines and this appears to have been mediated by the brown bag medication review. The intervention was not effective at reducing PIP and this was because GP did not identify PIP as intended and did not always act on identified PIP, see Supplementary Figure 2 for a summary.

### 4.2 Comparison with other literature

A culture of diagnosing and prescribing and a predominantly single disease focus of clinical guidelines have been identified as barriers to deprescribing.^21^ SPPiRE GPs reflected that participation in the intervention made them more confident in stopping medicines. Qualitative work describing GPs’ approaches to managing patients with multimorbidity has suggested that GPs are reticent to ‘rock the boat’ in these older complex patients.^22^ Similarly qualitative work with patients and their carers has suggested that both can be reluctant to stop a medicine that is not currently giving any perceived benefit for fear of missing out on possible future benefits.^23^ SPPiRE patients and GPs voiced similar views however most GPs felt patients were receptive to change and patients were generally positive in their description of medication changes that occurred during their SPPiRE review.

SPPiRE was not effective at reducing PIP, unlike the Data‐driven Quality Improvement in Primary care (DQIP) cluster RCT that showed that a GP‐oriented intervention comprising education, informatics and financial incentives was effective in improving the safety of prescribing.^24^ Results from the DQIP process evaluation suggested that the financial incentives may have been important for initiation of the intervention and for recruitment but were not considered an important ‘active ingredient’.^25^ Although SPPiRE practices received a small remuneration (€60 per patient recruited) to cover some of the practice costs for taking part, this was not part of the intervention and the use of financial incentives may have helped with the delays seen in initiating intervention implementation. Another effective component of the DQIP intervention that was important with initiation in larger practices, and was missing from SPPiRE, was discussion with the practices about potential practice processes to do the work.^25^ A final difference was that SPPiRE guided GPs in the identification of PIP but the DQIP tool identified and alerted GPs to PIP. As described previously, failure in identification of PIP by GPs may partially explain the lack of effect on this outcome measure in SPPiRE. There was a sustained effect from the DQIP intervention and their process evaluation suggested this may have been due to potential changes in the initiation of high‐risk prescribing.^26^ SPPiRE GPs also reported a better awareness of PIP and more caution about initiating certain classes of medicines after participating in the intervention.

SPPiRE was unique when compared with other recently published medicines management multimorbidity interventions in that it targeted a much more clinically vulnerable group with very significant polypharmacy and in that, no other health care professionals, aside from the GP, were involved in the intervention.^27^, ^28^, ^29^, ^30^, ^31^, ^32^, ^33^, ^34^ One of these studies, the 3D study, has published a process evaluation which showed that reach was lower than anticipated with a similar proportion to SPPiRE, having at least one review but only 49% having the full intervention (comprising a nurse and GP review).^35^ Similar to SPPiRE, staff shortage was the primary reason cited for this. Conversely to SPPiRE, the identification of patient priorities was the most consistently delivered component of the 3D intervention (99%).^35^ This may be because this was the first component of the review or because this process was delivered by the practice nurse. SPPiRE GPs suggested the involvement of the practice nurse and giving patients more time to reflect on their priorities may have better facilitated this process. This approach, where patients are sent a questionnaire and encouraged to consider their goals for treatment before the medication review, has been adopted for a similar intervention which is set in primary care in Canada and being evaluated by an individually randomized clinical trial.^36^ A similar intervention study set in primary care in Germany, where patients had a full 30 min interview with their GP to discuss priorities before a medication review, led to increased prescriptions for analgesics in the intervention group.^37^ Although pain was frequently identified as a priority for SPPiRE patients, this did not lead to the addition of new prescriptions for analgesics, this may be because the priority assessment process was not effective in addressing priorities or given the high number of medicines at baseline, pain was addressed by adjusting and augmenting current analgesic prescriptions. The most common medication change related to priority assessment in SPPiRE was a dose change (compared with stopping a medicine for the PIP assessment and brown bag processes), indicating that optimizing medications in polypharmacy is not just about changes in the number of medicines. A recently completed uncontrolled before‐after study of a similar patient‐centred medication review resulted in reduced medication regime complexity and enhanced adherence.^38^, ^39^ Future research in this area should focus on the development and evaluation of tools to assist both GPs and older people with multimorbidity in the identification of treatment priorities.

The PINCER trial demonstrated the clinical and cost‐effectiveness of pharmacist‐delivered medication reviews in primary care in the United Kingdom,^40^ and their process evaluation showed that GPs acted on pharmacist recommendations the majority of times (61%),^41^ compared with SPPiRE GPs who acted on self‐identified PIP 55% of the time. It may be that collaboration with a colleague facilitates making medication changes and a medicines management multimorbidity intervention that adopts this approach,^42^ has recently been piloted in Irish primary care.^43^ Primary care pharmacists are not a part of routine care in Ireland. However, given the context of GP shortage in Irish primary care a potentially more feasible approach may be an intervention that involves other health care professionals. Recently a small uncontrolled study based in Irish primary care has demonstrated the feasibility of pharmacist‐delivered medication reviews in primary care.^44^

### 4.3 Strengths and weaknesses

The aims of the SPPiRE process evaluation were pre‐specified and based on the MRC framework and a framework that was designed to guide the conduct of process evaluations for cluster RCTs.^14^ In addition, NPT was used to guide qualitative analysis and make sense of how the intervention was implemented. Triangulation of the qualitative and quantitative analyses allowed the results to be explored from different perspectives.^45^ Half of the GP interviews were carried out by the study manager who had significant contact with GPs during recruitment and intervention delivery increasing the likelihood of social desirability bias among interviewees. Having an independent, non‐medical interviewer may have reduced this bias, however GPs may have been more trusting or open with an interviewer they feel understands their perspective. The process evaluation was carried out parallel to the main trial, reducing the likelihood of bias, where investigators may be more focused on explaining the results rather than unearthing unintended consequences. The disadvantage of this approach was that we were unable to explore the reasons for the difference in PIP prevalence identified by the research pharmacist at baseline compared with intervention GPs during the medication review. Improvements in PIP in the control group during the study suggest that the identification of these patients on ≥15 medicines resulted in medication changes,^8^ and this may be an explanation but because the focus was on intervention implementation control practices were not interviewed. Finally, family and other informal caregivers are often involved in the management of medication for older people with multimorbidity,^46^ however as carers were not part of the trial, they were not invited for interview and their views were thus not included. The involvement of both formal and informal caregivers in the SPPiRE review process did not feature strongly in either GP or patient interviews and it may be that this was not sufficiently explored at the interview.

## 5 CONCLUSION

This mixed‐methods process evaluation showed that overall SPPiRE was implemented as planned in the majority of practice but that the context of resource and staff shortage affected implementation. In addition, deep‐seated patient and doctor views around medicines and medical decision‐making influenced adherence to the various subcomponents of the review. The SPPiRE intervention had a significant but small effect in reducing the number of medicines and this appears to have been mediated by the brown bag review element of the intervention. The intervention was not effective in reducing PIP and this may be because GPs were less familiar with the application of explicit prescribing criteria, especially given that the qualitative results indicated that GPs were motivated to identify and address PIP, although part of the lack of effect seen on PIP was due to improvements in the control group. A systems approach that is embedded in practice management software systems may be more effective, and override the need for the GP to identify PIP. The majority of participants had at least one priority identified and these were primarily symptom‐based. GPs’ views on this process varied considerably. Overall the majority of patients and GPs viewed SPPiRE positively, but adequate resourcing and integration into practice systems would be necessary for system‐wide implementation.

## AUTHOR CONTRIBUTIONS

Caroline McCarthy, Susan M. Smith, Barbara Clyne, Frank Moriarty, and Emma Wallace contributed to the study's conception and design. Material preparation, data collection and analysis were performed by Caroline McCarthy, Ivana Pericin and Bridget Kiely. The first draft of the manuscript was written by Caroline McCarthy and all authors commented on previous versions of the manuscript. All authors read and approved the final manuscript.

## CONFLICT OF INTEREST

The authors declare no conflict of interest.

## ETHICS STATEMENT

Ethical approval was granted by the Irish College of General Practitioners Research and Ethics Committee (SPPiRE). All recruited patients and GPs gave fully informed written consent.

## Supporting information

Supporting information.

Supporting information.

Supporting information.

Supporting information.

## Data Availability

The SPPiRE website usage data sets are available from the Zenodo repository (https://doi.org/10.5281/zenodo.6784724). Qualitative data that support the findings of this study are available on request from the corresponding author. The data are not publicly available due to privacy or ethical restrictions.
