# Supplementary material for: Patient and general practitioner experiences of implementing a medication review intervention in older people with multimorbidity: Process evaluation of the SPPiRE trial
Source: Health Expect. 2022 Oct 17;25(6):3225–37. doi: 10.1111/hex.13630 (PMC9700182; doi:10.1111/hex.13630)
Supplement: Supplementary file 1 — Supporting information. [file HEX-25--s001.docx]

Contents

[Appendix 1: SPPiRE PIP Criteria 2](#_Toc112857083)

[Appendix 2: SPPiRE Interview Topic Guide – Intervention GPs 4](#_Toc112857084)

[Appendix 3: SPPiRE Interview Topic Guide – Intervention Patients 6](#_Toc112857085)

[Appendix 4: Baseline characteristics of interviewees 7](#_Toc112857086)

[Appendix 5: Practice characteristics and SPPiRE intervention implementation 9](#_Toc112857087)

[Appendix 6: Difference in prevalence between baseline pharmacist and intervention GP PIP assessment 10](#_Toc112857088)

[Appendix 7: GP identified PIP and outcome action 11](#_Toc112857089)

[Appendix 8: Outcome of medication concerns identified during the brown bag review 12](#_Toc112857090)

## Appendix 1: SPPiRE PIP Criteria

| **Drug group** | **PIP** | **Reason** |
| --- | --- | --- |
| **Drug groups frequently associated with preventable drug related morbidity** | | |
| **NSAIDS** | with diuretic and ACEi/ARB (1) | Risk of renal impairment |
|  | with chronic kidney disease (eGFR <50) (1, 2) |  |
|  | for ≥ 12 weeks with no gastroprotection (1) | Risk of GI bleed |
|  | that is not COX 2 selective, with a history of PUD with no gastroprotection (2) |  |
|  | and antiplatelet with no gastroprotection (2) |  |
|  | with an anticoagulant (2, 3) |  |
|  | with severe hypertension or heart failure (2) | Risk of hypertension/ heart failure exacerbation |
|  | COX-2 selective with concurrent cardiovascular disease (2) | Increased risk of MI/CVA |
| **Antiplatelets** | and history of PUD with no gastroprotection (1, 3) | Risk of GI bleed |
|  | and anticoagulant with no gastroprotection (1, 3) |  |
|  | dual antiplatelet therapy with no gastroprotection (1) |  |
|  | consider intended duration of treatment if taking dual anti-platelet therapy for over one year post PCI (2) | Not usually indicated |
| **Anticoagulants** | for first uncomplicated DVT for >6 months duration (2) | Not indicated |
|  | for first uncomplicated PE for >12 months duration (2) |  |
|  | dabigatran (Pradaxa®) if eGFR <30 ml/min/ 1.73m^2^ or if renal function is unknown (2) | Risk of bleeding |
|  | rivaroxaban (Xarelto®)or apixaban (Eliquis®) if eGFR <15 ml/min/ 1.73m^2^ or if renal function is unknown (2) |  |
| **Diuretics** | and no U&E check in the last 48 weeks (1) | Risk of renal impairment and electrolyte abnormality |
|  | loop diuretic and thiazide diuretic and no U&E in the last 24 weeks (1) |  |
|  | loop diuretic for dependent oedema and no heart failure, liver failure or nephrotic syndrome (2) | Risks usually out-weigh benefits |
|  | thiazide diuretic with a history of gout (2) | Risk of precipitating gout |
| **Drugs groups associated with morbidity in the elderly** | | |
| **Anticholinergic drugs** | With comorbidities (3)  Dementia  Narrow angle glaucoma  Cardiac conduction abnormalities  Chronic prostatism | Exacerbation of co-morbidity |
|  | Concomitant use of two or more drugs with anticholinergic properties (2) | Risk of anticholinergic toxicity |
|  | tricyclic antidepressant as first line antidepressant (2) | Increased risk of adverse effects in older patients and alternatives available |
|  | antimuscarinic antihistamine (2) |  |
| **Benzodiazepines OR Z drugs** | for longer than 4 weeks (2) (1) | Risk of sedation, confusion, impaired balance, falls.  NNT 13 and NNH 6 when used for insomnia (4) |
| **Antipsychotics** | with dementia and no psychosis (1, 2) | Increased risk of stroke, only use when all other means have failed and shortest possible dose for shortest duration (5) |
| **Miscellaneous drug groups; included because of prevalence or high risk** | | |
| **Methotrexate** | not prescribed as weekly (1) | Increased risk of potentially fatal medication errors |
|  | prescribed > 1 strength tablet (1) |  |
| **Opioids** | used regularly with no laxative (2) | Risk of severe constipation |
| **Corticosteroids** | use ≥ 12 weeks with no bone protection (2) | Risk of fracture |
| **PPI** | for uncomplicated PUD/erosive peptic oesophagitis at full therapeutic dose ≥ 8 weeks (2) | Not indicated |
| **Metformin** | with eGFR < 30 ml/min/ 1.73m^2^ (2) | Risk of lactic acidosis |

1. Dreischulte T, Grant AM, McCowan C, McAnaw JJ, Guthrie B. Quality and safety of medication use in primary care: consensus validation of a new set of explicit medication assessment criteria and prioritisation of topics for improvement. BMC clinical pharmacology. 2012;12:5.

2. O'Mahony D, O'Sullivan D, Byrne S, O'Connor MN, Ryan C, Gallagher P. STOPP/START criteria for potentially inappropriate prescribing in older people: version 2. Age and ageing. 2015;44(2):213-8.

3. Clyne B, Bradley MC, Hughes CM, Clear D, McDonnell R, Williams D, et al. Addressing potentially inappropriate prescribing in older patients: development and pilot study of an intervention in primary care (the OPTI-SCRIPT study). BMC health services research. 2013;13:307.

4. Glass J, Lanctot KL, Herrmann N, Sproule BA, Busto UE. Sedative hypnotics in older people with insomnia: meta-analysis of risks and benefits. Bmj. 2005;331(7526):1169.

5. Ballard CG, Waite J, Birks J. Atypical antipsychotics for aggression and psychosis in Alzheimer's disease. Cochrane Database of Systematic Reviews. 2006(1).

## Appendix 2: SPPiRE Interview Topic Guide – Intervention GPs

**Prescribing for older patients**

- Can you talk me through how repeat prescriptions for older patients are reviewed in your practice?
  - Dedicated medication review visit Vs opportunistic
  - Who does it?
  - How are changes made when reviewing repeat prescriptions?
  - If problems are identified how are they managed, eg message left for patient/pharmacist through admin staff or GP contacting patient or written message left for patient with prescription?
- Can you describe some of the issues you face when reviewing prescriptions for these patient?
  - Uncertainty over who started the medicine and why
  - Uncertainty over potential benefits and risks
  - Reluctance to make too many changes due to fear of adverse consequences/the need for added visits
  - Patient preferences/demands
  - Previous positive or negative experiences of changing or discontinuing repeat medicines

**Summary of intervention use**

| **NPT Construct** | **Questions/Prompts** |
| --- | --- |
| **Coherence:** making sense of the intervention | What did you think would be the benefits of taking part in SPPiRE?  What were you wary of in terms of taking part?  How did you see these medication reviews fitting into your day to day practice? |
| **Cognitive Participation:** involvement with the intervention | Can you describe who else was from the practice was involved in the SPPiRE intervention. What do you feel their views of this were?  What did you see your role as being?  Describe how the practice has had to adapt to incorporate the implementation of the intervention.  Can you tell me what you thought about the training videos? |
| **Collective Action:** how practical work of *doing* the intervention is carried out within the organization | Can you talk me through the steps that were involved in performing the medication review?   - How were the appointments arranged, did the patients turn up? - Use of the SPPiRE website? - Was the review performed in one sitting? Was the patient present? - What worked well? What didn’t work so well? Was the website easy to use? - PIP identified? How did patient respond to suggested changes? - Brown bag - Patient priorities   Can you describe how compatible this was with existing practice? |
| **Reflexive Monitoring:** evaluation and appraisal of the intervention | Overall how would you describe your experience of taking part? Was it worthwhile? Would you like to see this or something similar in routine use (if yes, any suggestions as to what changes would be needed to improve intervention, if no reasons why it would be unfeasible in routine practice)?  What you would change?  In what way do you think it has had any impact?  Are there any aspects of the intervention that you have now incorporated into your routine practice?  Added workload, ADWEs? |

**Concluding comments**

- Overall is there anything else you would like to comment on?

## Appendix 3: SPPiRE Interview Topic Guide – Intervention Patients

**Medications in general**

- You were invited to take part in this study because of the number of medicines you are currently prescribed. How do you feel about the number of medicines you are prescribed?
  - What do you think is a lot of medicines to take?
  - How important/necessary do you feel they are?
  - Do you feel you know what medicines you are taking and why?
  - If you had a concern about your medicines, who would you talk to?
  - Do you feel you can talk to your GP about your medicines?
  - Do you like to be involved in decisions about your medicines?

**Summary of intervention use**

- As part of this study you would have been invited to attend a medication review visit with your GP.
  - What did you expect would happen?
  - Can you describe how that visit went?
  - Did you bring your medicines in with you to the visit?
  - How were your ideas, concerns and priorities addressed?
  - Were any changes made to your medicines? How did you feel about this?
  - Which things did you like the best about it? What did you not like/what would you change?
- What, if any, difference do you feel it has made to you
  - happier about medicines
  - reassured they were reviewed
  - concerns and priorities were addressed
  - left you feeling concerned or worried about your medicines
- Would you like to see something like this routinely used?
  - If yes, any suggestions are to how to improve or sustain it?
  - If no, why?

**Concluding comments**

Is there anything else the participant would like to add?

## Appendix 4: Baseline characteristics of interviewees

| **PATIENT INTERVIEWS** | | | | | | |
| --- | --- | --- | --- | --- | --- | --- |
| **ID** | **Gender** | **Age** | **Medication changes** | **Time** | **Interviewer** | **Follow-up questionnaire** |
| GP4P13 | Female | 73 | yes | 25.87 | CMC | yes |
| GP21P10 | Female | 67 | yes | 13.90 | CMC | yes |
| GP21P8 | Male | 75 | yes | 4.22 | CMC | yes |
| GP26P6 | Male | 75 | yes | 13.32 | CMC | yes |
| GP26P8 | Male | 68 | no | 16.12 | BC | yes |
| GP39P34 | Female | 73 | no | 8.53 | CMC | yes |
| GP39P41 | Female | 74 | no | 18.32 | CMC | yes |
| GP41P8 | Female | 67 | yes | 7.33 | BK | no |
| GP41P11 | Female | 82 | no | 9.03 | BK | yes |
| GP46P5 | Male | 72 | no | 8.42 | BK | no |
| GP46P7 | Female | 66 | yes | 11.75 | BK | no |
| GP50P1 | Female | 68 | no | 14.28 | BK | yes |
| GP50P8 | Male | 72 | yes | 11.33 | BK | yes |
| GP54P25 | Female | 84 | no | 5.37 | BK | yes |
| GP54P28 | Male | 70 | no | 7.18 | BK | no |
| GP61P6 | Male | 75 | no | 7.78 | BK | yes |
| GP61P8 | Male | 78 | no | 7.62 | BK | yes |
| GP68P2 | Male | 72 | yes | 13.22 | BK | no |
| GP68P4 | Female | 68 | yes | 8.37 | BK | yes |
| GP81P20 | Female | 76 | no | 8.32 | BK | no |
| GP81P26 | Male | 82 | no | 9.73 | BK | yes |
| GP97P7 | Male | 84 | no | 7.47 | BK | no |
| GP97P10 | Female | 84 | no | 16.93 | BK | yes |
| GP122P11 | Female | 76 | no | 9.85 | CMC | no |
| GP127P9 | Female | 82 | no | 6.57 | BK | yes |
| GP127P31 | Female | 67 | no | 3.98 | BK | yes |
| GP128P15 | Male | 71 | yes | 21.07 | CMC | yes |
|  |  |  |  |  |  |  |

| **GP INTERVIEWS** | | | | | |
| --- | --- | --- | --- | --- | --- |
| **ID** | **Gender** | **Role** | **Location** | **Time** | **Interviewer** |
| GP4 | male | GP partner | Rural | 27.08 | CMC |
| GP12 | female | GP partner | Urban | 18.17 | CMC |
| GP13 | male | GP assistant | Urban | 22.22 | CMC |
| GP21 | male | GP principal | Mixed | 22.02 | CMC |
| GP26 | female | GP partner | Urban | 25.20 | CMC |
| GP39 | male | GP partner | Mixed | 21.67 | CMC |
| GP41 | male | GP principal | Rural | 32.08 | BK |
| GP46 | male | GP partner | Urban | 11.68 | BK |
| GP54 | male | GP partner | Rural | 16.20 | BK |
| GP58 | male | GP partner | Mixed | 18.23 | BK |
| GP61 | female | GP assistant | Urban | 15.00 | BK |
| GP68 | male | GP partner | Urban | 18.37 | BK |
| GP81 | female | GP partner | Mixed | 22.50 | BK |
| GP97 | male | GP partner | Mixed | 11.25 | BK |
| GP122 | male | GP partner | Mixed | 18.67 | CMC |
| GP127 | female | GP partner | Urban | 12.47 | BK |
| GP137 | female | GP principal | Urban | 16.52 | CMC |
| GP128 | female | GP principal | Urban | 21.25 | CMC |

## Appendix 5: Practice characteristics and SPPiRE intervention implementation

| **GP ID** | **Practice size^¥^** | **Practice**  **Location** | **Reviewed/**  **Recruited** | **No. days late** | **PIP***  **N (%)** | **Concern^**  **N (%)** | **Priority^§^**  **N (%)** | **Meds stopped**  **(Mean)** |
| --- | --- | --- | --- | --- | --- | --- | --- | --- |
| 4 | 37 | Rural | 10/11 | 0 | 8 (80) | 9 (90) | 10 (100) | 4.89 |
| 12 | 35 | Urban | 9/10 | 0 | 9 (100) | 5 (56) | 9 (100) | 1.67 |
| 13 | 45 | Urban | 10/10 | 0 | 6 (60) | 2 (20) | 3 (30) | 0.10 |
| 21 | 9 | Mixed | 9/9 | 100 | 7 (78) | 0 | 3 (33) | 0.50 |
| 26 | 28 | Urban | 7/7 | 0 | 6 (86) | 4 (57) | 7 (100) | 1.50 |
| 39 | 54 | Mixed | 5/9 | 317 | 4 (80) | 2 (40) | 3 (60) | 2.25 |
| 41 | 8 | Rural | 8/8 | 0 | 6 (75) | 7 (88) | 8 (100) | 1.25 |
| 46 | 21 | Urban | 5/5 | 0 | 5 (100) | 1 (20) | 3 (60) | 1.60 |
| 49 | 34.5 | Mixed | 0/5 | N/A | N/A | N/A | N/A | N/A |
| 50 | 53 | Urban | 8/9 | 3 | 5 (63) | 7 (88) | 7 (88) | 1.56 |
| 54 | 30 | Rural | 10/11 | 243 | 10 (100) | 7 (70) | 8 (80) | 2.60 |
| 58 | 29 | Mixed | 6/6 | 0 | 5 (83) | 5 (83) | 5 (83) | 1.33 |
| 61 | 18 | Urban | 8/8 | 330 | 6 (75) | 4 (50) | 8 (100) | 0.13 |
| 68 | 14 | Urban | 8/8 | 0 | 7 (88) | 3 (38) | 8 (100) | 6.57 |
| 73 | 88 | Urban | 0/8 | N/A | N/A | N/A | N/A | N/A |
| 81 | 43 | Mixed |  | 0 | 5 (63) | 5 (63) | 3 (38) | 1.75 |
| 97 | 10 | Mixed | 10/10 | 110 | 10 (100) | 4 (40) | 8 (80) | 0.20 |
| 103 | 22 | Mixed | 1/5 | 266 | 1 (100) | 1 (100) | 1 (100) | 5.00 |
| 115 | 16 | Urban | 0/7 | N/A | N/A | N/A | N/A | N/A |
| 116 | 34 | Urban | 12/15 | 298 | 10 (83) | 12 (100) | 12 (100) | 0.42 |
| 117 | 24 | Urban | 3/7 | 12 | 3 (100) | 1 (33) | 3 (100) | 0.67 |
| 122 | 40 | Mixed | 4/5 | 5 | 3 (75) | 3 (75) | 4 (100) | 3.00 |
| 127 | 30 | Urban | 9/10 | 14 | 5 (56) | 7 (78) | 6 (67) | 1.05 |
| 128 | 12.5 | Urban | 5/5 | 0 | 3 (60) | 4 (80) | 2 (40) | 1.20 |
| 137 | 24 | Urban | 3/5 | 0 | 1 (33) | 2 (67) | 2 (67) | 2.50 |
| 138 | 32 | Rural | 5/6 | 30 | 5 (100) | 0 (0) | 5 (100) | 1.20 |

*Practices that declined or did not respond to telephone interview invitations are highlighted in grey.*

*^¥^ Measured by the number of GP sessions per week, whereby one session is one half day (either a morning or afternoon clinic).*

** The number of participants and proportion of those reviewed, with at least 1 PIP identified.*

*^ The number of participants and proportion of those reviewed, with at least 1 concern identified.*

*^§^ The number of participants and proportion of those reviewed, with at least 1 priority identified.*

## Appendix 6: Difference in prevalence between baseline pharmacist and intervention GP PIP assessment

| **PIP** | **GP**  **N=163 (%)** | **Trial pharmacist**  **N=208 (%)** |
| --- | --- | --- |
| The use of a benzodiazepine or z drug for longer than 4 weeks | 56 (34.4) | 86 (41.3) |
| Full dose PPI for longer than 8 weeks | 56 (34.4) | 126 (60.6) |
| Regular opioid with no laxative | 28 (17.2) | 39 (18.8) |
| Therapeutic duplication | 27 (16.6) | 40 (19.2) |
| Anticholinergic with co-morbidities | 19 (11.7) | 24 (11.5) |
| Two or more anticholinergic drugs | 17 (10.4) | 71 (34.1) |
| Loop diuretic for dependent oedema | 16 (9.8) | 46 (22.1) |
| Any diuretic use and no RP in past 48 weeks | 10 (6.1) | 34 (16.3) |
| NSAID with diuretic and ACEi | 9 (5.5) | 8 (3.8) |
| Loop and thiazide diuretic and no RP in last 24 weeks | 5 (3.1) | 3 (1.4) |

*Abbreviations: PIP: potentially inappropriate prescription, PPI; proton pump inhibitor, RP; renal profile, NSAID; Non-steroidal anti-inflammatory drug, ACEi; Angiotensin converting enzyme inhibitor.*

## Appendix 7: GP identified PIP and outcome action

| **PIP** | **N** | **Outcome** | | | | | |
| --- | --- | --- | --- | --- | --- | --- | --- |
|  |  | **Drug stopped** | **Dose change** | **Blood test** | **No change** | **No data^1^** | **Other^2^** |
| Full dose PPI >8 weeks | 60 | 10 | 27 | 0 | 14 | 6 | 3 |
| BZO/Z drug >4 weeks | 58 | 6 | 13 | 0 | 31 | 4 | 4 |
| Opioid, no laxative | 29 | 2 | 4 | 0 | 9 | 2 | 12 |
| Therapeutic duplication | 27 | 10 | 0 | 0 | 6 | 4 | 7 |
| Anticholinergic with co-morbidity | 20 | 3 | 1 | 0 | 10 | 3 | 3 |
| Two or more anticholinergics | 17 | 7 | 1 | 0 | 5 | 2 | 2 |
| Loop diuretic for dependent oedema | 16 | 7 | 1 | 2 | 3 | 2 | 1 |
| Diuretic, no RP >48 weeks | 11 | 2 | 1 | 6 | 0 | 2 | 0 |
| NSAID, diuretic and ACEi | 9 | 5 | 0 | 1 | 1 | 0 | 2 |
| Loop and thiazide diuretic, no RP >24 weeks | 5 | 2 | 0 | 2 | 0 | 0 | 1 |
| TCA first line | 5 | 2 | 0 | 0 | 1 | 2 | 0 |
| Steroid >12 weeks, no bone protection | 4 | 0 | 0 | 0 | 1 | 0 | 3 |
| NSAID >12 weeks, no PPI | 3 | 0 | 0 | 0 | 0 | 1 | 2 |
| NSAID with anticoagulant | 3 | 1 | 0 | 0 | 2 | 0 | 0 |
| DAPT no PPI | 3 | 1 | 0 | 0 | 1 | 0 | 1 |
| Thiazide diuretic and gout | 3 | 0 | 2 | 0 | 1 | 0 | 0 |
| Antipsychotics with dementia | 3 | 0 | 1 | 0 | 2 | 0 | 0 |
| NSAID with CKD | 2 | 0 | 1 | 0 | 0 | 0 | 1 |
| DAPT >1 year | 2 | 1 | 0 | 0 | 0 | 1 | 0 |
| First generation antihistamine | 2 | 1 | 0 | 0 | 0 | 1 | 0 |
| MTX prescribed >1 strength tablet | 2 | 0 | 1 | 0 | 1 | 0 | 0 |
| COX-2 with CVD | 1 | 0 | 0 | 0 | 0 | 1 | 0 |
| NSAID, antiplatelet, no PPI | 1 | 0 | 0 | 0 | 0 | 0 | 1 |
| NSAID, PUD, no PPI | 1 | 0 | 0 | 0 | 0 | 1 | 0 |
| Antiplatelet, PUD, no PPI | 1 | 0 | 0 | 0 | 0 | 0 | 1 |
| Antiplatelet, anticoagulant | 1 | 0 | 0 | 0 | 0 | 0 | 1 |
| Metformin, eGFR <30 | 1 | 1 | 0 | 0 | 0 | 0 | 0 |
| MTX, no FBC >12 weeks | 1 | 0 | 0 | 0 | 1 | 0 | 0 |
| Total | 291 | 61  (21%) | 53  (18%) | 11  (4%) | 89  (31%) | 32  (11%) | 45  (15%) |

*^1^GP did not fill out action data for PIP*

*^2^Either specialist referral, medicine added or multiple medication changes*

*Abbreviations: PIP; potentially inappropriate prescription, PPI; proton pump inhibitor, BZO; benzodiazepine, RP; renal profile, NSAID; Non-steroidal anti-inflammatory drug, ACEi; Angiotensin converting enzyme inhibitor, TCA; tricyclic antidepressant, DAPT; dual antiplatelet therapy, CKD; chronic kidney disease, MTX; methotrexate, PUD; peptic ulcer disease, FBC; full blood count*

## Appendix 8: Outcome of medication concerns identified during the brown bag review
