# Supplementary figures and images for: Patient and general practitioner experiences of implementing a medication review intervention in older people with multimorbidity: Process evaluation of the SPPiRE trial
Source: Health Expect. 2022 Oct 17;25(6):3225–37. doi: 10.1111/hex.13630 (PMC9700182; doi:10.1111/hex.13630)

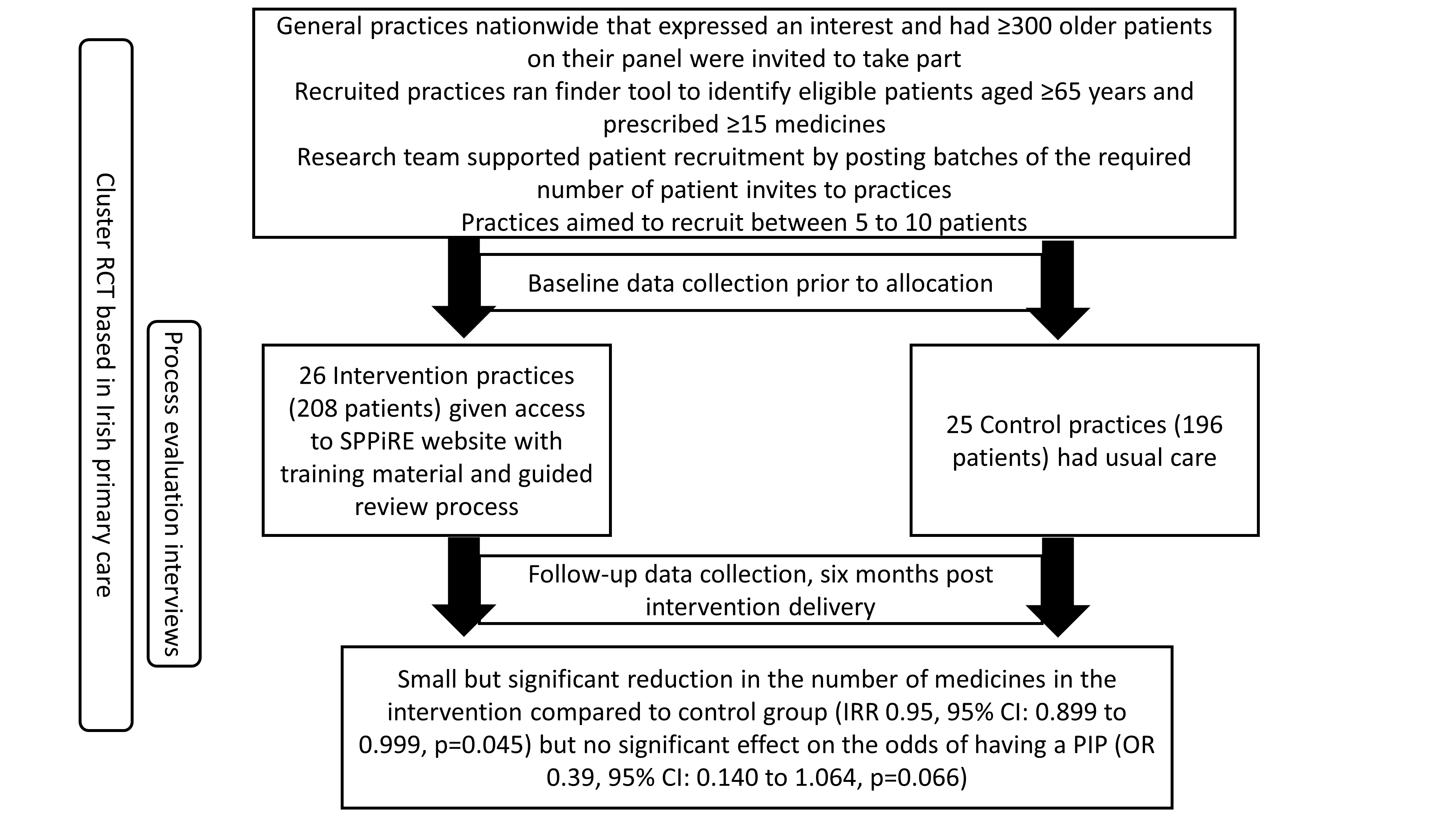

Supplement: Supplementary file 2 — Supporting information. [file HEX-25--s002.tif]

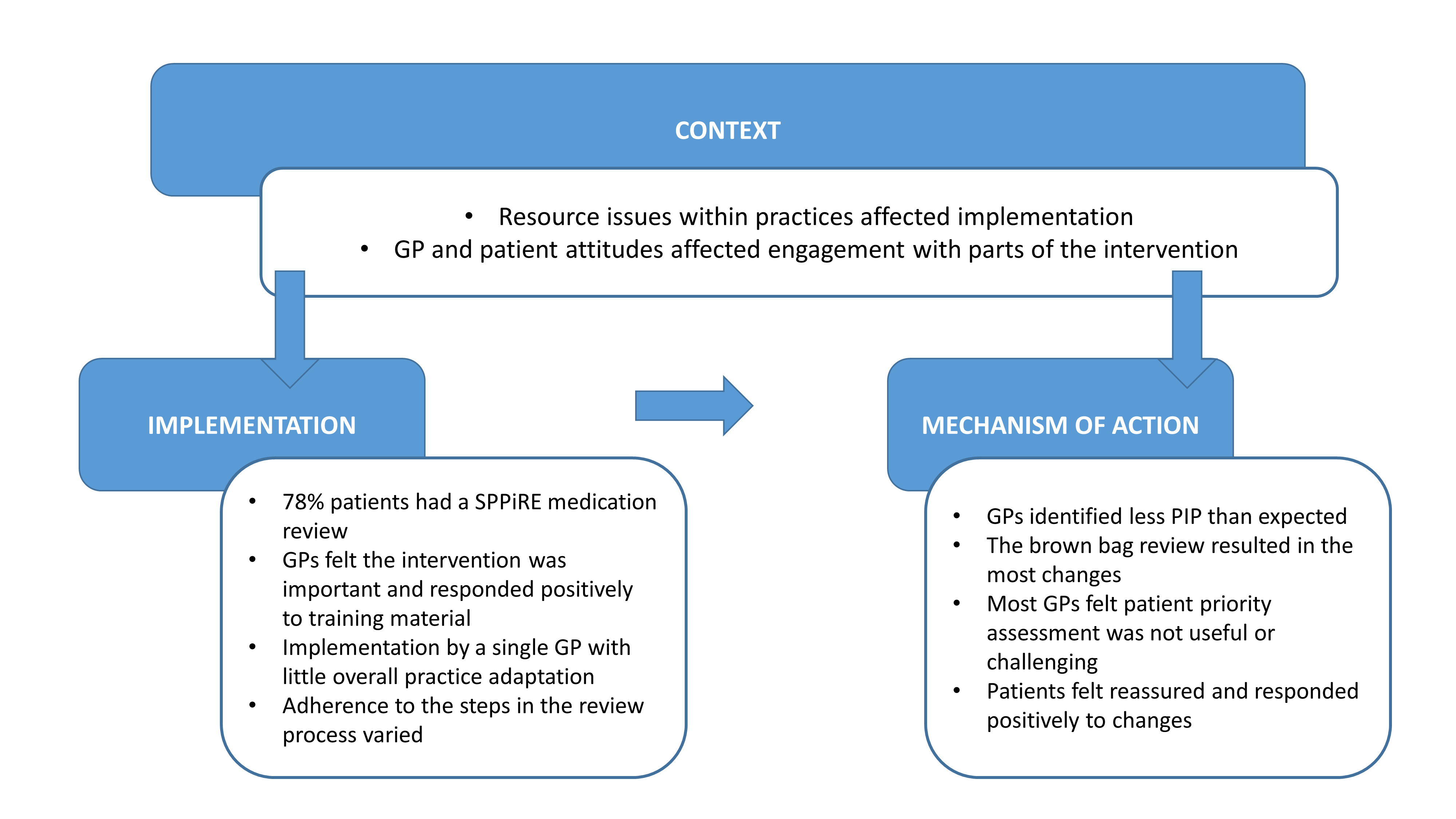

Supplement: Supplementary file 3 — Supporting information. [file HEX-25--s004.tif]
