# Supplementary material for: Patient and general practitioner experiences of implementing a medication review intervention in older people with multimorbidity: Process evaluation of the SPPiRE trial
Source: Health Expect. 2022 Oct 17;25(6):3225–37. doi: 10.1111/hex.13630 (PMC9700182; doi:10.1111/hex.13630)
Supplement: Supplementary file 4 — Supporting information. [file HEX-25--s003.docx]

Supplementary Figure 1 Methods and results of SPPiRE cluster RCT

Supplementary Figure 2 Summary of SPPiRE process evaluation results

Summary of context, implementation and mechanism of action of the SPPiRE intervention [1, 2]

1. Kyne K, McCarthy C, Kiely B, Smith SM, Clyne B. Study protocol for a process evaluation of a cluster randomised controlled trial to reduce potentially inappropriate prescribing and polypharmacy in patients with multimorbidity in Irish primary care (SPPiRE). *HRB open research*. 2019;2:20.

2. Moore GF, Audrey S, Barker M, Bond L, Bonell C, Hardeman W, et al. Process evaluation of complex interventions: Medical Research Council guidance. *BMJ (Clinical research ed).* 2015;350:h1258.
